# Spatial and Temporal Epidemiology of Lumpy Skin Disease in the Middle East, 2012–2015

**DOI:** 10.3389/fvets.2016.00019

**Published:** 2016-03-03

**Authors:** Mohammad A. Alkhamis, Kimberly VanderWaal

**Affiliations:** ^1^Environmental and Life Sciences Research Center, Kuwait Institute for Scientific Research, Kuwait City, Kuwait; ^2^Department of Veterinary Population Medicine, College of Veterinary Medicine, University of Minnesota, St. Paul, MN, USA

**Keywords:** lumpy skin disease virus, Middle East, ecological niche modeling, time-dependent reproductive number, surveillance

## Abstract

Lumpy skin disease virus (LSDV) is an infectious disease of cattle that can have severe economic implications. New LSD outbreaks are currently circulating in the Middle East (ME). Since 2012, severe outbreaks were reported in cattle across the region. Characterizing the spatial and temporal dynamics of LSDV in cattle populations is prerequisite for guiding successful surveillance and control efforts at a regional level in the ME. Here, we aim to model the ecological niche of LSDV and identify epidemic progression patterns over the course of the epidemic. We analyzed publically available outbreak data from the ME for the period 2012–2015 using presence-only maximum entropy ecological niche modeling and the time-dependent method for the estimation of the effective reproductive number (R-TD). High-risk areas (probability >0.60) for LSDV identified by ecological niche modeling included parts of many northeastern ME countries, though Israel and Turkey were estimated to be the most suitable locations for occurrence of LSDV outbreaks. The most important environmental predictors that contributed to the ecological niche of LSDV included annual precipitation, land cover, mean diurnal range, type of livestock production system, and global livestock densities. Average monthly effective R-TD was equal to 2.2 (95% CI: 1.2, 3.5), whereas the largest R-TD was estimated in Israel (R-TD = 22.2, 95 CI: 15.2, 31.5) in September 2013, which indicated that the demographic and environmental conditions during this period were suitable to LSDV super-spreading events. The sharp drop of Isreal’s inferred R-TD in the following month reflected the success of their 2013 vaccination campaign in controlling the disease. Our results identified areas in which underreporting of LSDV outbreaks may have occurred. More epidemiological information related to cattle populations are needed to further improve the inferred spatial and temporal characteristics of currently circulating LSDV. However, the methodology presented here may be useful in guiding the design of risk-based surveillance and control programs in the region as well as aid in the formulation of epidemic preparedness plans in neighboring LSDV-free countries.

## Introduction

Lumpy Skin Disease virus (LSDV) is in the genus *Capripoxvirus* and family *Poxviridae* and is the causal agent of Lumpy Skin Disease (LSD), a transmissible disease of cattle with significant economic implications (1, 2). The disease is characterized by large skin nodules covering the entire body of the animal, emaciation, poor milk production, and abortion. The severity of the clinical symptoms varies from acute to subclinical forms (1–3). Due to its observed economic impacts on the global cattle industry (4, 5), the World Organization of Animal Health (OIE) has classified LSDV as a notifiable disease. LSDV is mainly transmitted via arthropod vectors. LSDV outbreaks are typically associated with wet and warm seasons (6) and mosquitoes such as *Aedes aegypti* are efficient mechanical vector for the transmission and maintenance of LSDV (7). Direct and indirect contact between infected and susceptible animals is not considered to be a pathway for transmission (8). The virus can infect other small ruminants such as sheep and goats, but does not cause clinical disease (9, 10). However, contact of cattle herds with sheep and goat in grazing/watering areas has been implicated as a potential risk factor for mechanical transmission of LSDV (11).

Clinical cases consistent with LSDV were first observed in northern Zambia in 1929 (12). In the 1940s, the disease rapidly spread to cattle populations of other southern African countries (5). Until 1984, LSDV was maintained within the countries of sub-Sahara Africa, where its pathogenicity increased over time leading to severe pandemics (5, 13). In the Middle East (ME), unconfirmed cases of the disease were reported in Oman and Kuwait between 1984 and 1988 (14–16). However, the first confirmed LSDV cases within ME countries were reported in Egypt in 1988 (15). In 1989, the first confirmed transcontinental spread of LSDV from African to Asian ME countries occurred when the disease was reported in Israel (17). This transcontinental spread of LSDV was attributed to wind-borne transmission via stable flies (*Stomoxys calcitrans*) from Egypt (17). During the same year, suspected cases of LSDV were also reported in Saudi Arabia in a herd of Arabian oryx (*Oryx leucoryx*) (18). Further outbreaks were also reported in Kuwait, Bahrain, Yemen, United Arab Emirates, and Sudan (15, 16, 18, 19). LSDV was reintroduced into Egypt via imported cattle from the African horn countries in 2006 (5, 20). Subsequently, LSDV cases were again reported in Israel, Bahrain, Oman, and the West Bank (20). For the first time, confirmed cases of LSDV were reported in Lebanon, Jordan, and Turkey between 2012 and 2013 (21). The Syrian Arab Republic has been implicated in the introduction of LSDV into Turkey (20). Due to the current armed conflict, the disease is believed to be underreported in Syria. This situation has raised major concerns in the international community, as the disease may spread into LSDV-free European member state countries using Turkey as a portal of introduction (22). Most recently, new cases of LSDV have been reported in Iran, Azerbaijan, Iraq, Greece, and Cyprus between 2014 and 2015 (19, 23, 24).

Control and prevention of LSDV in ME countries is largely dependent on the infrastructure of their veterinary services. Implementation of vaccination, movement restrictions, and stamping out policies were successful in eradicating the disease in Egypt and Israel during 2006 (5, 17, 19, 25). However, in other ME countries, the combination of unstable political situations, uncontrolled animal movements, lack of laboratory diagnostic resources, and inadequate communication with international health organizations are considered major causes for the failure of any efforts to control or prevent the spread of highly infectious animal diseases like LSDV (5, 26).

Due to the important role of blood-feeding arthropods in the transmission of LSDV, its spread and geographical distribution are heavily influenced by environmental conditions (27–29). Hence, ecological niche models provide a tool to extract associations between environmental factors (e.g., climate and land cover) and outbreak occurrence data, use those associations to characterize the environmental requirements for the disease agent and vector, and subsequently project those associations to predict the geographic distribution of LSDV in underreporting regions (30). Modeling the temporal and spatial dynamics of LSDV will provide a robust platform for guiding the design of infectious disease surveillance systems in the ME, and subsequently improve control and prevention. Furthermore, such knowledge will shed further insights into the epidemiology of LSDV in the ME and assess the risk of introduction into disease-free countries like the European Union member states.

Attempts to model the spatial and temporal epidemiology of LSDV have been minimal globally and non-existent in regions like the ME. Thus, the ultimate goal of this study is to characterize the spatial and temporal dynamics of LSDV in ME countries. Here, we tested whether environmental and demographic variables can predict the geographic distribution of recent LSDV outbreaks reported in cattle populations of the ME for the period 2012–2015 using a presence-only maximum entropy ecological niche modeling method (Maxent). Furthermore, we estimated multiple effective reproductive numbers to assess transmission potential and efficacy of control and prevention measures during the course of the epidemic in the region. Results of such methodologies may shed further insights into the spatial and temporal epidemiology of LSDV in the ME. Subsequently, these results may contribute to the formulation of surveillance programs that selectively target high-risk cattle areas with specific demographic and environmental factors in the ME region and guide epidemic preparedness efforts in neighboring LSDV-free countries.

## Materials and Methods

### Data Source

**Figure 1:**
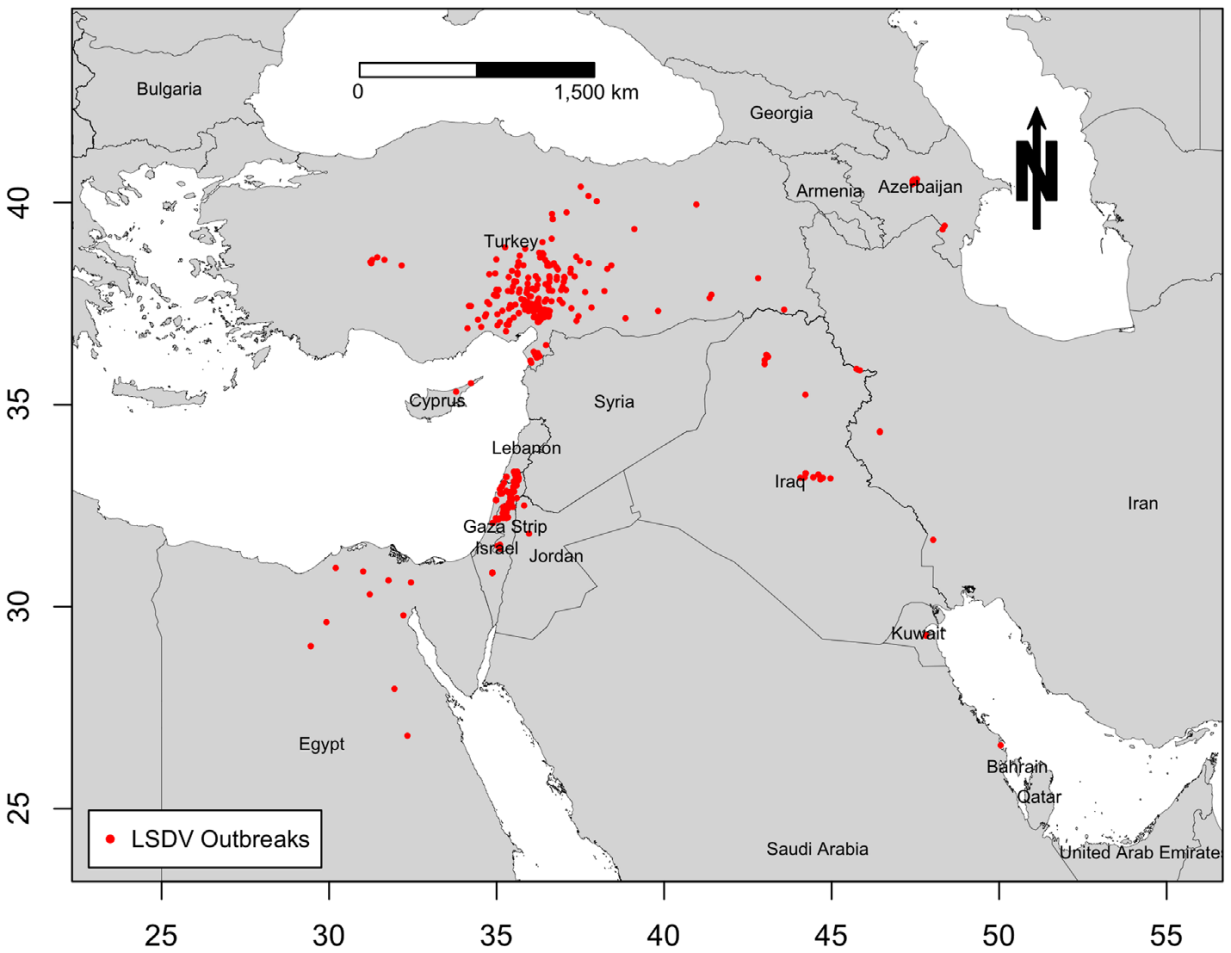
**Geographic distribution of lumpy skin disease outbreaks reported in cattle in the Middle East from July 2012 through May 2015**.

Middle East countries in this study include those of southwest Asia and northeast Africa, excluding Afghanistan, Pakistan, and India. Thus, the study region includes Iran, Turkey, Syria, Lebanon, Israel, Palestinian Territories, Jordan, Iraq, Egypt, Libya, Sudan, Djibouti, Eritrea, Somalia, and countries of the Arabian Peninsula (Saudi Arabia, Yemen, Oman, United Arab Emirates, Qatar, Bahrain, Kuwait). Azerbaijan and Cyprus were also included in this study, because they were severely impacted during LSDV epidemics, and they both actively exchange cattle with neighboring ME countries. We retrieved the outbreak data used for this study from the Food and Agriculture Organization of the United Nation (FAO) Global Animal Disease Information System EMPRES-i (31), which included all geographic locations (Figure 1) and onset dates of 604 LSDV outbreaks in cattle populations reported by ME countries to the OIE from July 2012 to May 2015. Due to unavailability of information regarding the population at risk, reported outbreaks, defined as the detection of one or more cases of the disease, are considered the epidemiological units. Reported outbreaks usually included a group of cattle herds that were epidemiologically linked, and thus could be considered part of the same outbreak event. Since 3 years is the time frame of the present study, we choose months as a suitable time unit for summarizing the epidemic curve of LSDV outbreaks in the ME. Our temporal aggregation unit has been chosen to ensure that at least one outbreak was reported at any point on the epidemic curve and to fulfill the requirements of the subsequent analyses.

For environmental variables (predictors) for the ecological niche of LSDV in the ME, we selected climate variables, density of four livestock species (cattle, buffalo, sheep, and goat), global land cover, and global livestock production system. We obtained climate raster data (5 km^2^ resolution) from the WorldClim website,^1^ a commonly used interpolated global climate data resource for ecological modeling and GIS (32). WorldClim is a set of global climate data layers (climate grids) with a spatial resolution of 1, 5, 9, or 18 km^2^. Variables included in the analysis were monthly mean, minimum and maximum temperature, monthly precipitation, and altitude (Table S1 in Supplementary Material). Those climatic data are further derived into a series of 19 bioclimatic variables (Table S1 in Supplementary Material). The WorldClim variables are smoothed maps of mean monthly climate data obtained from a variety of sources from 1950 through 2000. Data have been interpolated down to a 30 arc-second high-resolution grid, which is often referred to as “1 km^2^” resolution. Due to the large geographical range of the current study, only bioclimatic variables with an approximate spatial resolution of 5 km^2^ were used to maintain the computation intensity of the models and constant cell size with other environmental layers. Thus, the total number of climatic predictors used in the ecological niche modeling process sums to 24 variables (32).

We retrieved estimated livestock density raster data for cattle, buffalo, sheep, and goats with an approximate spatial resolution of 5 km^2^ (Table S1 in Supplementary Material) from the FAO GeoNetwork webpage (33). These livestock density rasters were estimated based on observed livestock statistics (number of animals/km^2^) at different administrative levels and their relationship with various environmental variables such as land suitable for livestock production (excluding water surfaces and protected areas) (33). Similarly, we retrieved the global livestock production systems grid from the above data source (34) to provide an indirect estimate of the geographic distribution of each production system, which are defined based on livestock animal densities within a premise in a given geographical region of specific climatic conditions and intensification level (Table S1 in Supplementary Material). This data grid classifies those production systems into 14 different geographic features (Table S2 in Supplementary Material). Finally, we obtained an estimate of the geographical distribution of 16 different land cover features in the ME (Table S1 in Supplementary Material), including forests, croplands, grasslands, urban, etc., by using the high spatial resolution (0.5 km^2^) land cover data grid (Table S2 in Supplementary Material), known as MODIS-based global land cover climatology, from the United States geographical survey (USGS) webpage^2^ (35). Thus, our final raster dataset compromised of 30 environmental covariates (Table S2 in Supplementary Material).

We converted all of the above environmental data layers into a common projection and map extent using the Raster package (36) implemented in R statistical software version 3 (37). We cropped each raster so that the geographical extent of the subsequent spatial analyses covered only the ME region. Furthermore, because the land cover data were at different spatial scale (0.5 km^2^) from other environmental variables (5 km^2^), we aggregated and resampled raster data to create a uniform grid size, which resulted in a scale of approximately 6 km^2^. Furthermore, we examined the environmental data for collinearity by visually inspecting the relation between pairs of variables in scatter-plots. Finally, we transformed the locations of LSDV outbreaks into a smoothed kernel density grid raster with 5 km^2^ spatial resolution and a search radius of 10 km^2^, and calculated the pairwise Spearman’s correlation coefficients between each environmental variable (with exception of land cover and livestock production system, as these are not continuous variables) and the geographical distribution of LSDV outbreaks for the Maxent model. All LSDV outbreaks reported in the ME were included in the subsequent analyses.

### Ecological Niche Modeling

We predicted the risk of LSDV in the ME using the presence-only maximum entropy ecological niche modeling technique (Maxent) (38), where risk is defined as the probability that a geographic location is suitable for the occurrence of LSDV. The Maxent program version 3.3.3 was implemented as a function in Dismo package in R. A detailed description of the Maxent algorithm is available elsewhere (38). Briefly, Maxent builds ecological niche models by extracting associations between presence data (e.g., LSDV outbreaks) and environmental variables, using those associations to characterize the environmental requirements for the disease agent, and subsequently deploying those associations to predict suitable geographical locations in non-sampled areas. In this study, we used the default convergence threshold, regularization, and number of iterations. In addition, we used the default logistic model to ensure that predictions gave estimates between 0 and 1 for the risk of outbreak per map cell. Initially, we fitted separate Maxent models for each of the climatic variables and their association with LSDV outbreaks, a procedure that resembles a bivariate analysis. We then selected variables that had greater than 10% relative contribution in the prediction (improvement in predictive power relative to a null model) and included them in the subsequent multivariable models, along with the above non-climatic demographic variables. Cattle density was used as an indirect estimate of the distribution of the population at risk, which we included as background sampling data, while other environmental variables were included as direct predictors for the risk of LSDV (39). We set the Maxent program to randomly sample 10,000 locations within the ME to form background data (i.e., weights) for LSDV outbreaks in cattle. The established background data do not attempt to guess at the absence of LSDV locations (i.e., negative LSDV locations) but rather characterizes the nature of the cattle density distribution in the ME. In this sense, the background data establish the environmental domain of the study region, while LSDV outbreak presence data establish under which conditions an outbreak is more likely to be present than on average.

We evaluated the performance of the candidate Maxent models by partitioning the outbreak data into training and testing sets and using the threshold independent method on each partitioned set, which characterizes the performance of the model across the full range of possible probability thresholds for presence/absence predictions (38). We used *k*-fold method as a partitioning scheme (40), which randomly samples the data with replacement and creates *k* partitions, where each candidate Maxent model was tested five times (*k* = 5 in this study) against 1000 randomly generated background points (pseudo-absences). For the threshold independent method, we calculated the area under the curve (AUC) through a receiver operator characteristic (ROC) plot of the sensitivity (the proportion of true predicted known presences, known as omission error) vs. 1 – specificity (proportion of false predicted known absences, known as commission error) over the whole range of threshold values between 0 and 1. The training set (training AUC) was used for model building, and the test set (testing AUC) was used to evaluate model accuracy using the value of the AUC. The AUC value ranges from 0.5 (entirely random predictive model) to a maximum value of 1 (perfectly discriminating predictive model). Maxent models with AUC >0.75 for both training and testing data are usually considered accurate (41). We used the jackknife tests to calculate the contribution of each environmental variable to the final model’s prediction. Because of the large geographic area analyzed in this study, we used a calibrated AUC (cAUC) for the final Maxent model to evaluate the presence of the spatial sorting bias (SSB) as suggested elsewhere (42). If the cAUC value was close to 1, then one can conclude the absence of SSB (i.e., countries with high reported outbreak incidences have small impact on the resulted Maxent model), whereas if the value was close to zero, then SSB is present in the data (i.e., countries with high reported outbreak incidences have large impact on the resulted Maxent model). cAUC is commonly smaller than training or testing AUCs because it uses a randomly selected smaller subset of the presence data (42).

Because presence data were largely skewed toward Israel and Turkey, we repeated the above analyses three times to validate the adequacy of the selected environmental data in predicting the probability of the spatial distribution of LSDV in the ME. Model I was fit with all reported outbreaks in the ME; Data for Model II comprised only of outbreaks reported in Israel and the West Bank; Model III was fit only with outbreaks reported in Turkey; Model IV was fit with outbreaks reported in the ME excluding Israel, West Bank and Turkey. We compared the magnitude of change in the AUC values described above to further assess the sensitivity of the Maxent model to the reporting bias in the presence data (38, 39, 41, 42). We used the same approach to inspect for confounding of one environmental variable to another in the each ecological niche model by removing variables that contribute more than 10% in the prediction of probability for the spatial distribution of LSDV. Thus, if the AUC values changed by more than 5%, it would indicate that the variable is an important confounding factor. Finally, we tested the adequacy of the risk map generated by model II (Maxent model predicted by the observed Israeli outbreaks) in predicting the spatial distribution of LSDV in Turkey (i.e., predicted probabilities based on the observed outbreaks reported in Turkey). Hence, we created a “validation Maxent” model that compromised of the observed Turkish outbreaks, as independent presence-only data, and the risk map generated by Model II (Israeli Maxent model) as a single predictor to test whether the AUC values (training and testing AUC values) were less than 0.75. If the AUC values were less than 0.75, then the Israeli model is an inaccurate predictor of LSDV risk in Turkey.

### Estimation of the Time-Dependent Reproductive Numbers

We used a likelihood-based procedure to estimate effective time-dependent reproductive numbers (R-TDs) for each outbreak from the observed epidemic curve of LSDV, as suggested elsewhere (43). The method has been implemented in the R package “R0” within the software environment (44). First, we aggregated the outbreak data by month to ensure that at least one outbreak was reported per time unit. Second, we identified the serial interval distribution of the generation time from the time lag between consecutive reported outbreaks and estimated its mean and SD from the observed epidemic curve. Third, we estimated the R-TD for each outbreak as the sum of the probabilities that a given outbreak was the source of infection for subsequent outbreaks based on elapsed time (44). Essentially, this method is based on averaging over all transmission networks compatible with the epidemic curve during the course of the epidemic. 95% confidence intervals (CI) were obtained through simulations, as described elsewhere (44).

## Results

### LSDV Occurrence and Epidemic Curve

**Figure 2:**
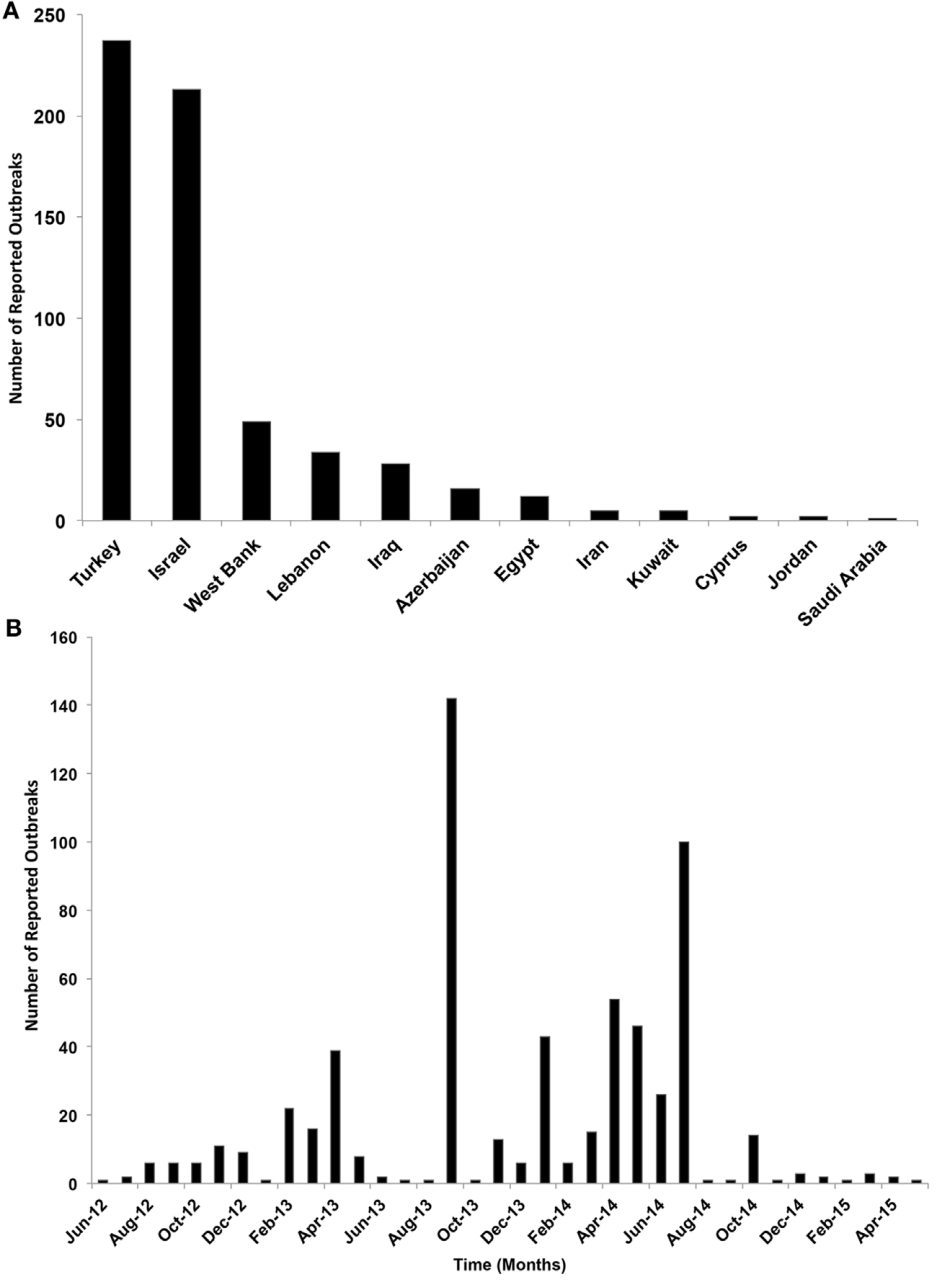
**(A)** Number of reported lumpy skin disease outbreaks (per country) in cattle in the Middle East from July 2012 through May 2015. **(B)** Temporal distribution of lumpy skin disease outbreaks (per month) in cattle in the Middle East from July 2012 through May 2015.

Only 12 ME countries reported LSDV outbreaks in cattle between 2012 and 2015. The proportion of LSDV outbreaks occurring in Turkey and Israel (including West Bank) and Lebanon and Iraq was substantially greater than in other locations, whereas in Cyprus, Egypt, Iran, Saudi Arabia, Lebanon, Kuwait, and Jordan did not exceed 3% of reported outbreaks (Figure 2A). During the course of the epidemic, the highest numbers of detected outbreaks in the ME countries were reported in September 2013 in Israel followed by July 2014 in Turkey (Figure 2B, Figure S1 in Supplementary Material).

### Ecological Niche Model

Pairwise Spearman correlations indicated that our selected environmental predictors were significantly associated with the geographical distribution of LSDV in the ME (Table 1). The least significant correlation (*r* = 0.015) was observed with buffalo density (Table 1). Our final Maxent model indicated that only five of the selected environmental variables were needed to adequately predict the geographical risk of LSDV outbreaks in the ME with an AUC higher than 0.75, and a cAUC closer to 1 than 0 (Table 2). The final Maxent model included annual precipitation and mean diurnal temperature range as climatic predictors, type of livestock production system, land cover, and goat, sheep, and buffalo densities as demographic variables (Table 2; Model I). Land cover and annual precipitation were found to be the most important environmental predictors for LSDV in Israel and West Bank (Model II), while annual precipitation and mean diurnal temperature range were found to be the most important environmental predictors for LSDV in Turkey (Model III). However, Livestock production system and goat density were found to be the most important environmental predictors for LSDV in other ME countries (Model IV). As expected, removing variables that contributed more than 10% to the prediction resulted in more than 5% change in the AUC estimates for each ecological niche model, which suggests that those variables were indeed important confounders.

**Table 1:** **Spearman correlation coefficients (*r*) between each environmental variable and geographical distribution of lumpy skin disease outbreaks in cattle in the Middle East**.

	Geographical distribution of LSDV in cattle (*r*)	*P*-value
Annual precipitation	0.53	<0.001
Mean diurnal temperature range	−0.36	0.012
Goat density	−0.52	0.031
Buffalo density	0.015	0.243
Sheep density	0.35	0.052
Cattle density	0.45	<0.001

**Table 2:** **Estimates of relative contributions of the environmental variables to each Maxent model and their validation AUCs values**.

Variable	% Contribution	Training data AUC^a^	Test data AUC **±** SD^b^	cAUC^c^ **±** SD
**Model I = All ME countries**				
Annual precipitation	36.1	0.92	0.91 ± 0.18	0.81 ± 0.22
Livestock production system	14.9			
Mean diurnal temperature range	14.2			
Goat density	14.1			
Land cover	13.3			
Sheep density	4.3			
Buffalo density	3.1			
**Model II = Israel**				
Land cover	51.3	0.95	0.93 ± 0.09	0.68 ± 0.08
Annual precipitation	33.9			
Livestock production system	7.7			
Goat density	5.9			
Mean diurnal temperature range	3.1			
Sheep density	0.6			
Buffalo density	0.6			
**Model III = Turkey**				
		0.90	0.88 ± 0.04	0.73 ± 0.11
Annual precipitation	29.5			
Mean diurnal temperature range	21			
Sheep density	19.1			
Goat density	15.7			
Buffalo density	7.5			
Land cover	4			
Livestock production system	3.3			
**Model IV = Other ME countries excluding Israel and Turkey**				
Livestock production system	44.5	0.93	0.93 ± 0.13	0.75 ± 0.17
Goat density	22.3			
Mean diurnal temperature range	12.3			
Sheep density	11.5			
Annual precipitation	4.0			
Buffalo density	3.8			
Land cover	1.6			

*Model I includes all reported outbreaks in the Middle East; Model II includes only reported outbreaks in Israel and West Bank; Model III includes only reported outbreaks in Turkey; Model IV includes reported outbreaks in the Middle East with the exception of Israel, West Bank and Turkey*.

*^a^Area under the curve*.

*^b^SD for the test and calibrated AUC*.

*^c^Calibrated AUC for test data*.

**Figure 3:**
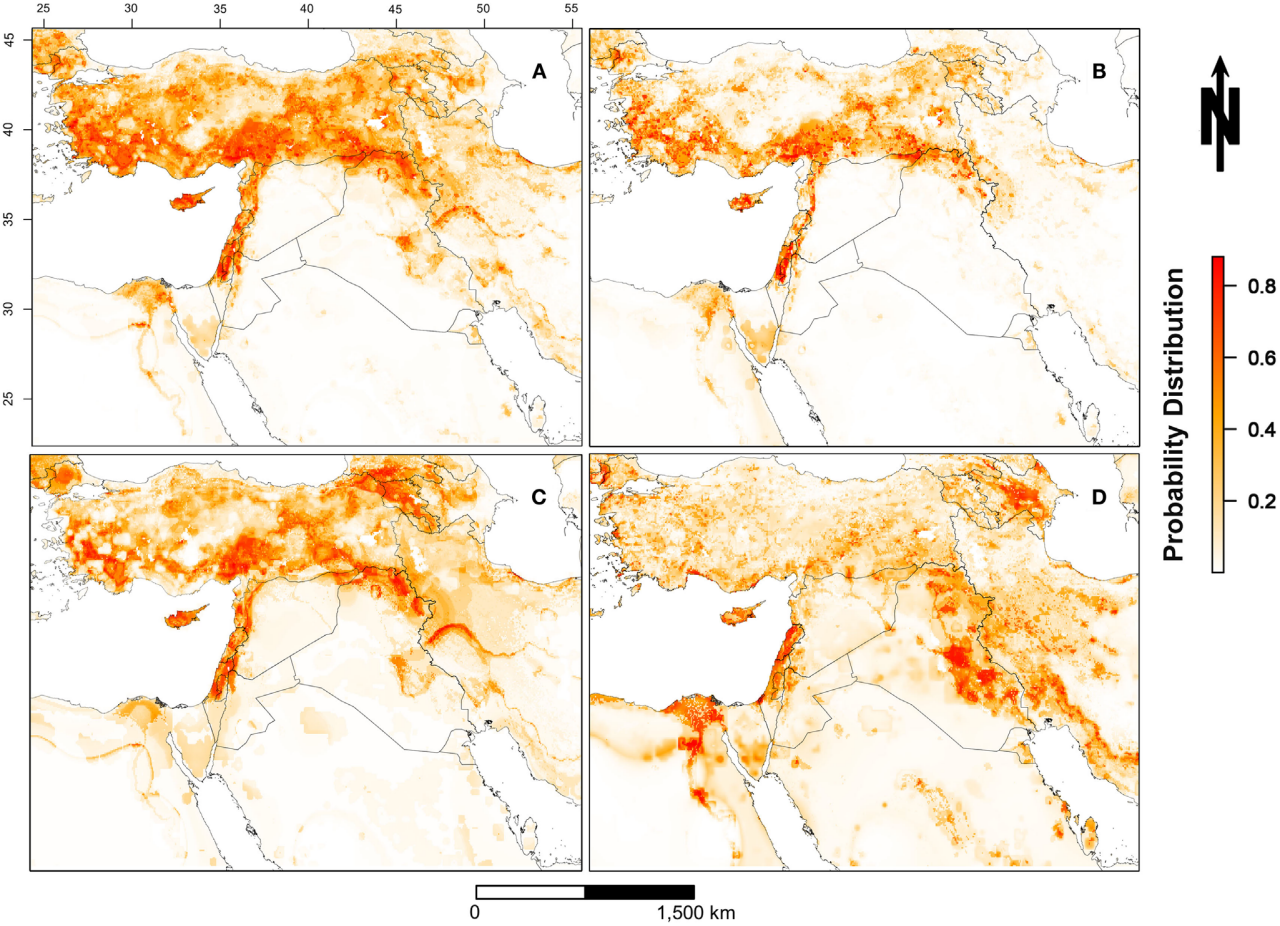
**Probability of spatial distribution of lumpy skin disease virus (LSDV) in cattle in the Middle East from July 2012 through May 2015**. [**(A)** = Model I] includes all reported outbreaks in the Middle East; [**(B)** = Model II] includes only reported outbreaks in Israel and West Bank; [**(C)** = Model III] includes only reported outbreaks in Turkey; [**(D)** = Model IV] includes reported outbreaks in the Middle East with the exception of Israel, West Bank, and Turkey. The legend on the bottom right represents the probability distribution for the most suitable locations for LSDV in cattle (red >0.8 and white <0.2).

**Figure 4:**
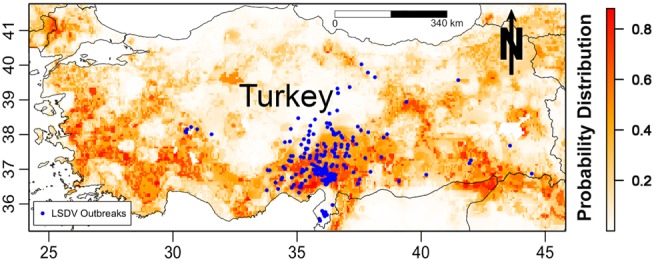
**Probability of spatial distribution of lumpy skin disease virus (LSDV) in cattle in Turkey predicted by the Israeli Maxent model (II)**. The figure is a magnified snap shot for Turkey in Figure 3B. The blue dots represent the observed geographical distribution of LSDV in Turkey.

The predicted spatial distribution for suitable areas for LSDV in cattle (i.e., areas with high probability of having conditions promoting transmission) in the ME is shown in Figure 3. Results of all four Maxent models indicate that the selected environmental variables were adequate predictors for the risk of LSDV in the ME (i.e., suitable areas for the introduction of LSDV based on AUC values), with no substantial changes in the magnitude of the estimated AUCs across models. High-risk areas (probability >0.6) for all Maxent models (Models I–IV, Figures 3A–D) were consistently identified in northern and central Israel, Cyprus, Lebanon, northwest Syria, the southern border of Turkey, and northern Iraq. Furthermore, additional risk areas with less consistent magnitudes of risk across Maxent models (probability between 0.3 and 0.6) were identified in Azerbaijan, Georgia, Kuwait, Egyptian Nile Valley, and northern Iran. However, substantial discrepancies were observed in the identified risk areas between Maxent models I–III and Maxent Model IV. Unlike the first three Maxent models (Models I–III, Figures 3A–C), Maxent Model IV identified the eastern Iraq and western Iran as high risk-areas (probability >0.6) for LSDV outbreaks (Figure 3D). In addition, Maxent Models I–III (Figures 3A–C) inferred more widespread risk of LSDV across Turkey when compared to the findings of Maxent Model IV (Figure 3D). The observed outbreaks reported in Turkey were completely encompassed by the high-risk areas predicted by Model II (Figure 4), where the AUC values of the model were equal to 0.79 and 0.78 (±0.09 SD) for training and testing sets (Turkish observed outbreaks), respectively.

Generally, the range of annual precipitation for the ME region, estimated by the bioclimatic variables, was between 0 and 235 mm, while the mean diurnal temperature range was between 5.5 and 18°C. Results of the final Maxent models (Model I–VI) indicated that geographical regions with approximate annual precipitation between 50 and 100 mm were found most suitable for the risk of LSDV in cattle (probability >0.6), and geographic regions with mean temperature variations of approximately 12°C between day and night were found most suitable for the risk of LSDV. Geographic regions with croplands were found most suitable for LSDV. Urban and mixed rain-fed arid livestock production areas were also found to be suitable for the disease. Finally, geographical regions with low goat density were found to be suitable for the risk of LSDV. However, discrepancies in the magnitude of relative contributions of the environmental predictors were observed across Maxent models (Table 2). Annual precipitation was consistently an important predictor for Models I, II and III (relative contribution >29%), while the type of Livestock production system was consistently an important predictor for models I and IV (relative contribution >14%; Table 2). Land cover, mean diurnal temperature range, and Goat Density were also within the top two most important predictors for Model II, III, and IV, respectively (Table 2).

### Epidemic Time-Dependent Reproductive Number

**Figure 5:**
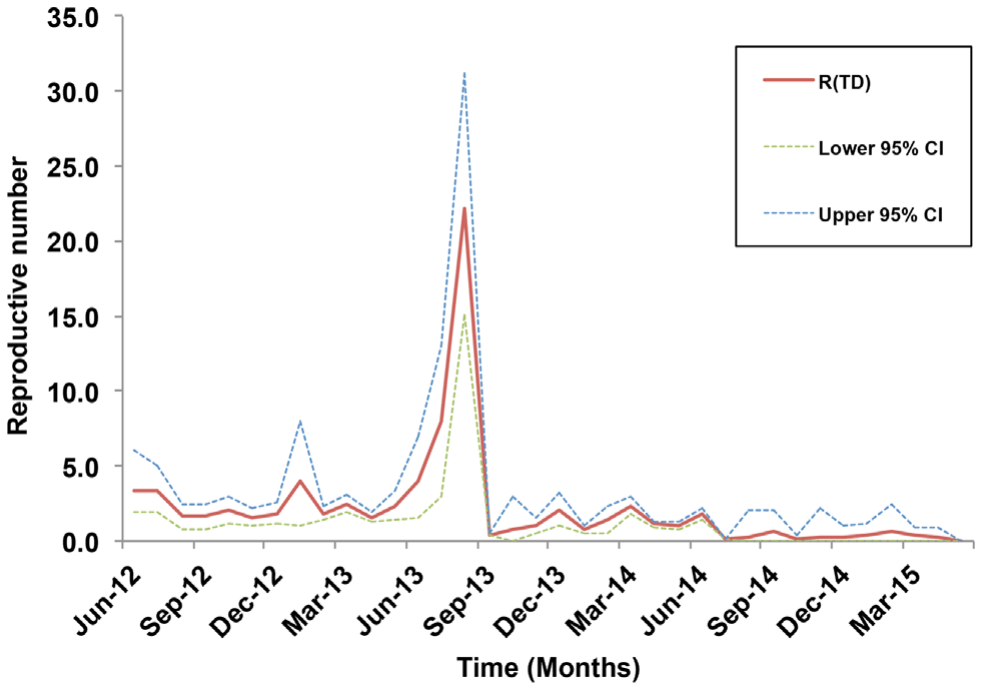
**Estimates of the time-dependent reproductive numbers and their 95% confidence internals (CI) over the course of the lumpy skin disease epidemic in the Middle East from July 2012 through May 2015**.

Our data covered 36 months of outbreaks for the currently circulating LSDV (Figure 2B). We converted the epidemic curve into the time course of effective R-TDs. No seasonal patterns were observed in our epidemic curve. Instead, LSDV outbreaks followed were erratic in ME countries (Figure 2B). Our estimates indicate that the average monthly effective R-TD was 2.2, indicating a more than twofold increase in the epidemic size during the course of epidemic between 2012 and 2015 (RT-D = 2.2; 95% CI: 1.2, 3.5) (Figure 5). However, the largest R-TD was estimated in Israel (R-TD = 22.2, 95% CI: 15.2, 31.5) in September 2013, followed by a substantial drop in the estimate (0.4, 95% CI: 0.3, 0.5) the following month (Figure 5).

## Discussion

This study represents a novel approach to model the spatial and temporal dynamics of LSDV in ME countries. First, we used ecological niche modeling, based on presence-only outbreak data, to reveal spatial variation in risk of LSDV and the environmental factors underlying this pattern. Second, we estimated multiple effective reproductive numbers during the course of the epidemic to assess spread and transmission potential of LSDV within ME countries.

We investigated the role of environmental variables in the development of an ecological niche model for LSDV in cattle independently of country boundaries to gain a regional picture of LSDV risk across the ME. During the course of the current epidemic, the highest reported incidence of LSDV outbreaks was observed in Turkey and Israel (including the West Bank), and therefore, both countries were predicted as the highest risk areas for the disease (Figure 3). By using Maxent models to understand environmental factors underpinning high-risk areas, our results predict that high-risk areas also occur in northeastern and northwestern Syria, Cyprus, Lebanon, and the whole northern region of Iraq based on sharing similar environmental characteristics with areas of high reported incidence (Figure 3A). These high-risk areas have conditions suitable for transmission and circulation of LSDV in cattle populations, even if LSDV has not yet been reported. Hence, our results suggest that the identified geographic regions or countries with few reported LSDV outbreaks are still at high risk. Our AUC values suggest that the selected environmental variables of our ecological niche modeling approach were adequate predictors for the LSDV outbreaks in cattle in the ME between 2012 and 2015 (Table 2).

Our ecological niche modeling approach identified annual precipitation and mean diurnal range (out of 24 climatic variables) as the most important climatic predictors for LSDV risk in cattle in the ME during the course of the current epidemic (Tables 1 and 2). Indeed, such weather conditions, which often occur in early and late summer and spring seasons, are characteristic of northern ME countries located in the Eurasian continent as compared to countries of the Arabian Peninsula. This result agrees with the notion that regions with wet and warm climatic conditions are prime habitat for blood-feeding arthropods, which are the main vectors for LSDV transmission and spread (5, 19, 20, 29, 45).

Types of land cover and livestock production system are important predictors of risk for circulation of LSDV (Tables 1 and 2). Croplands and urban and mixed rain-fed arid livestock production systems are quite common in northern parts of the ME countries, especially in Egypt and Israel, where LSDV outbreaks frequently occurred (34, 45). These findings agree with past LSDV studies in Ethiopia, in which the characteristics of cattle farms, grazing system, and land cover features were important risk factors for LSDV in the country (4, 11, 28, 46). Furthermore, cattle farms with close geographic proximity to urban areas are quite common in ME countries including countries of the Arabian Peninsula, where severe LSDV outbreaks were recently reported (24, 45). This could be attributed to multiple risk factors including: favorable climatic conditions for vectors, low biosecurity measures, introduction of new animals and mixing of different livestock species, as suggested elsewhere (5, 13, 20, 26). In addition, cattle farms with close proximity to urban areas are under intensive human surveillance, and thus the number of detected outbreaks is expected to be higher than non-urban production systems.

Interestingly, our final Maxent model suggests that areas with low-density sheep and goat populations were suitable for the transmission of LSDV (Table 2). This is not surprising, since nomadic and free grazing sheep and goat herds are common in ME countries. Contact with small cattle farms and/or herds is frequent. Furthermore, small-scale farms with mixed livestock species are also common in ME countries (26). Frequent contact of cattle herds with small nomadic sheep and goat herds has been considered as a risk factor for the transmission of LSDV, as described elsewhere (11, 46). Therefore, obtaining detailed information related to farm characteristics, such as vaccination status, biosecurity level, size, type of production, and species, might substantially improve the prediction of our presented ecological niche model.

Although our selected environmental variables were adequate predictors (based on AUC values) of LSDV outbreaks in the ME, dissimilarities were observed in the spatial distributions of risk (Figure 3) and relative contribution of each predictor (Table 2). Indeed, this is expected due the diverse nature of each environmental predictor in each country or geographical areas within the ME. For example, Maxent model II suggested that the combination of land cover and livestock production system represented by proximity to croplands and mixed rain-fed arid livestock production areas, respectively, constituted ideal environments for the spread and maintenance of LSDV during wet seasons in Israel and other counties with similar environmental conditions. However, model III strongly suggested that climate and densities of sheep and goat populations played significant roles in the spread and maintenance of LSDV in Turkey and countries with similar environmental conditions (Table 2), which explains the expansion of the high-risk areas across that country (Figures 3A,C). Finally, the complex combination of climatic conditions, livestock densities, land cover, and production system features played a significant role in the transmission and spread of LSDV in the Egyptian Nile valley, eastern and central Iraq, and western Iran (Figure 3D).

It is important to note that the dissimilarities between the risk maps for the four scenarios (Figures 3A–D) are mainly attributed to the magnitude of correlation between the selected environmental predictors and the outbreak data used for each scenario. This highlights the influence and potential biases of the input dataset in determining environmental risk factors in Maxent models. When using such approaches to infer spatial patterns of infection risk, it is important to remember that there is no single “true” model predicting risk across all contexts. Indeed, environmental factors contributing to risk may differ across space and time. However, our approach of using multiple input datasets both acknowledges that results may differ according to the input dataset and also allows for the identification of spatial and environmental patterns that are consistent regardless of the input dataset. This provides a robust way to address issues emerging from reporting biases across countries.

Our inferred average effective R-TD, on a regional scale, suggested a fairly large epidemic size with a 2.2-fold increase (RT-D >1) in the number of outbreaks during its course between 2012 and 2015 in the ME. Furthermore, estimates of the R-TD before September 2013 were on average larger than those estimated after that month (Figure 5). Although severe outbreaks were reported after September 2013, this is most likely attributed to the continuous increase in the political instability of the region after the Arab Spring. Thus, apparent reductions in numbers of outbreaks may be due to substantial underreporting rather than successful implementation of control measures, with the exception of Israel. Underreporting of cases and lack of information on the time of implementation of control measures might substantially bias the estimates of the effective R-TD, which is a major limitation described elsewhere (43). We believe that the largest R-TD estimated for the epidemic in Israel is indicative of a super-spreading event that occurred during that period. Following this, Israel rigorously implemented control activities, including a massive national vaccination campaign implement in 2013 (47), which led to the sharp drop in R-TD in the following month. Our study confirms that the demographic and environmental characteristics of Israel made it a suitable geographic area for super-spreading events of LSDV (inferred by large R-TD values), and the sharp drop of R-TD after its initial spike may reflect that their strong veterinary infrastructure and the 2013 vaccination campaign had successfully controlled this severe outbreak (47). Similar conclusions can be inferred in Turkey, where a large number of outbreaks were also reported after September 2013. Indeed, despite being environmentally suitable for LSDV, Turkey’s low R-TDs could be attributed to effective control and prevention measures (24).

Reporting bias may not only bias the estimates of the effective R-TD, but it can also substantially bias the results of ecological niche analysis. For example, it has been shown that reporting bias has implications for the selection of a representative ecological niche model (42). In this study, reporting bias had a significant role in the discrepancies between the four Maxent models in terms of the distribution of spatial risk (Figure 3) and relative contribution of each environmental predictor (Table 2). Although reporting of LSDV is mandatory for OIE-member countries, this mandate does not necessarily imply that every case or outbreak has been reported, arguably, because of substantial difference in the surveillance capabilities between different ME countries. For that reason, outbreak data used in our study may have been biased toward countries with higher surveillance capabilities, leading to predictions skewed toward these countries, as in the case of Israel and Turkey. To address this, we also constructed a Maxent model that excluded data from high-reporting regions (Turkey, Isreal, and the West Bank). A visual inspection of Figures 3A–D (model excluding data from high-reporting regions) reveals that the distribution of high-risk areas are similarly predicted by models built with or without data from high-reporting countries, though the absolute magnitude of risk differs somewhat. This gives us confidence that the overall Maxent model and predicted risk distributions are not skewed toward high-reporting countries.

Accuracy of the environmental variables in predicting the presence of the outbreaks might be substantially improved with more representative environmental data. In this study, we used the predicted global densities of different livestock species as a proxy for real current densities. Therefore, using representative livestock densities specifically for our study region might substantially alter the results Maxent models and/or might affect the value of the percent contribution of each environmental predicator in each Maxent model (41). Unfortunately, getting representative accurate outbreak occurrence (including a standard definition of an outbreak) and environmental data is almost impossible due to political unrest in the region and the discrete nature of ME countries in sharing their information.

That said, the use of ecological niche models might compensate for under reporting bias because predictions are based on the correlation between environmental predictors and disease in mostly areas where high concentrations of data available. These associations between environmental factors and disease occurrence can then be used to predict risk in areas where data maybe scarce. For example, it has been speculated that Syria may be a source of LSDV for the rest of the region, as described elsewhere (20), and that cases in Syria may be severely underreported due to armed conflicts. Despite the paucity of data from Syria, our analysis confirms that areas within Syria are suitable for the spread of LSDV (Figures 3A–D). Furthermore, our rigorous validation methods, including the use of the Israeli risk map to directly and independently predict the risk of LSDV in Turkey, indicated that the Israeli model is an accurate predictor of risk outside Isreal’s borders (Figure 4). Thus, we suggest that the selected environmental layers identified in countries with high reporting can be used to adequately predict the risk of LSDV throughout the ME and can compensate for the reporting bias suffered by the observed data.

Although, our identified environmental predictors match those identified in the past published literature, as described above, the resulting risk maps for LSDV occurrence are not definitive and need to be updated periodically as new data emerges. Thus, in the event of future epidemics, these analyses need to be repeated and refined in order to be subsequently used in surveillance, control, and prevention strategies. Furthermore, the model predictions suggest that the reason why some ME countries did not report cases of LSDV might not be due to underreporting, but simply because they did not exhibit suitable environmental conditions for the spread of LSDV in cattle. Therefore, an additional use of ecological modeling here may be to distinguish between geographic areas in which absence of reporting was likely due to a true absence of disease and those in which disease may be underreported but present. It is important to note that our analytical approach neither attempts to predict future epidemics, in terms of where and when the next LSDV outbreak will likely occur, nor provide definitive risk estimates for the introduction or occurrence of the outbreaks. Instead, our inferences are mainly useful in providing further insights into the current epidemiological situation of LSDV in the ME based on available data. Such knowledge might be useful in guiding the design of risk-based surveillance activities, in which sampling schemes can be targeted toward high-risk geographical areas and periods of time, identified by our analytical approach, and accordingly mobilize control, and prevention resources for the current LSDV epidemic.

## Conclusion

We modeled the spatial and temporal dynamics of LSDV in the ME in 2012–2015 using publically available outbreak data. We inferred significant epidemic transmission patterns in time for LSDV, and identified environmental factors that shape the ecological niche of the disease in cattle in the ME between 2012 and 2015. Furthermore, most of the significant R-TDs (>1), which have reached up to 22.2-fold increase, indicated that the identified environmental conditions in Israel and the Palestinian territories were conducive for region-level super-spreading events of LSDV outbreaks and that Isreal’s 2013 vaccination campaign was indeed successful in controlling the disease. While our results identified areas in which underreporting of LSDV outbreaks may have occurred, they also suggest that more epidemiological information related to cattle populations is needed to further improve the inferred spatial and temporal characteristics of currently circulating LSDV. However, the methodology presented here may contribute to the formulation of risk-based surveillance programs, with targeted sampling schemes, that selectively target high-risk cattle areas with specific demographic and environmental factors in the ME region and guide epidemic preparedness efforts in neighboring LSDV-free countries.

## Author Contributions

MA formulated the spatial and temporal models and was primarily responsible for report and manuscript preparation; KV provided interpretation on the use of epidemiological models, collaborated in the design of the analytical model, and assisted in manuscript preparation.

## Conflict of Interest Statement

The authors declare that the research was conducted in the absence of any commercial or financial relationships that could be construed as a potential conflict of interest.

## Footnotes

^1^http://www.worldclim.org

^2^http://landcover.usgs.gov
