# Supplementary material for: Spatial and Temporal Epidemiology of Lumpy Skin Disease in the Middle East, 2012–2015
Source: Front Vet Sci. 2016 Mar 3;3:19. doi: 10.3389/fvets.2016.00019 (PMC4776163; doi:10.3389/fvets.2016.00019)
Supplement: Supplementary file 1 [file data_sheet_1.docx]

Supplementary Material

Spatial and Temporal Epidemiology of Lumpy Skin Disease in the Middle East, 2012-2015

Mohammad A. Alkhamis * and Kimberly VanderWaal

*** Correspondence:** A. Alkhamis, Environmental and Life Sciences Research Center, Kuwait Institute for Scientific Research, P.O. Box 24885, Safat 13109, Kuwait. mkhamis@kisr.edu.kw

**Supplementary Table 1.** Data sources and properties of the enviromental covariates used to model the probability of the spatial distribution of lumpy skin disease outbreaks reported in the Middle East.

| **ID** | **Source** | **Type** | **Time period** | **Spatial resolution** |
| --- | --- | --- | --- | --- |
| 1 | WorldClim Global Climate Data | Minimum temperature | 1950-2000 | 5 km^2^ |
| 2 |  | Maximum temperature |  |  |
| 3 |  | Mean temperature |  |  |
| 4 |  | Precipitation |  |  |
| 5 |  | Altitude |  |  |
| 6 |  | BIO1 = Annual Mean Temperature |  |  |
| 7 |  | BIO2 = Mean Diurnal Range (Mean of monthly (max temp - min temp)) |  |  |
| 8 |  | BIO3 = Isothermality (BIO2/BIO7) (* 100) |  |  |
| 9 |  | BIO4 = Temperature Seasonality (standard deviation *100) |  |  |
| 10 |  | BIO5 = Max Temperature of Warmest Month |  |  |
| 11 |  | BIO6 = Min Temperature of Coldest Month |  |  |
| 12 |  | BIO7 = Temperature Annual Range (BIO5-BIO6) |  |  |
| 13 |  | BIO8 = Mean Temperature of Wettest Quarter |  |  |
| 14 |  | BIO9 = Mean Temperature of Driest Quarter |  |  |
| 15 |  | BIO10 = Mean Temperature of Warmest Quarter |  |  |
| 16 |  | BIO11 = Mean Temperature of Coldest Quarter |  |  |
| 17 |  | BIO12 = Annual Precipitation |  |  |
| 18 |  | BIO13 = Precipitation of Wettest Month |  |  |
| 19 |  | BIO14 = Precipitation of Driest Month |  |  |
| 20 |  | BIO15 = Precipitation Seasonality (Coefficient of Variation) |  |  |
| 21 |  | BIO16 = Precipitation of Wettest Quarter |  |  |
| 22 |  | BIO17 = Precipitation of Driest Quarter |  |  |
| 23 |  | BIO18 = Precipitation of Warmest Quarter |  |  |
| 24 |  | BIO19 = Precipitation of Coldest Quarter |  |  |
| 25 | FAO GeoNetwork | Global cattle density | 2005 |  |
| 26 |  | Global buffalo density |  |  |
| 27 |  | Global goat density |  |  |
| 28 |  | Global sheep density |  |  |
| 29 |  | Livestock production systems with 14 discrete spatial features | 2011 |  |
| 30 | MODIS-based Global Land Cover | Land cover with 16 discrete spatial features | 2014 | 0.5 km^2^ |

**Supplementary Table 2.** Feature defintions and their assigned values for land cover and and livestock production systems.

| **Value** | **Feature definition** |
| --- | --- |
| ***Land cover*** | |
| 0 | Water |
| 1 | Evergreen Needle leaf Forest |
| 2 | Evergreen Broadleaf Forest |
| 3 | Deciduous Needle leaf Forest |
| 4 | Deciduous Broadleaf Forest |
| 5 | Mixed Forests |
| 6 | Closed Shrublands |
| 7 | Open Shrublands |
| 8 | Woody Savannas |
| 9 | Savannas |
| 10 | Grasslands |
| 11 | Permanent Wetland |
| 12 | Croplands |
| 13 | Urban and Built-Up |
| 14 | Cropland/Natural Vegetation Mosaic |
| 15 | Snow and Ice |
| 16 | Barren or Sparsely Vegetated |
| ***Livestock production systems*** | |
| 1 | Rangelands Hyperarid |
| 2 | Rangelands Arid |
| 3 | Rangelands Humid |
| 4 | Rangelands Temperate |
| 5 | Mixed Rainfed Hyperarid |
| 6 | Mixed Rainfed Arid |
| 7 | Mixed Rainfed Humid |
| 8 | Mixed Rainfed Temperate |
| 9 | Mixed Irrigated Hyperarid |
| 10 | Mixed Irrigated Arid |
| 11 | Mixed Irrigated Humid |
| 12 | Mixed Irrigated Temperate |
| 13 | Urban |
| 14 | Other |

**
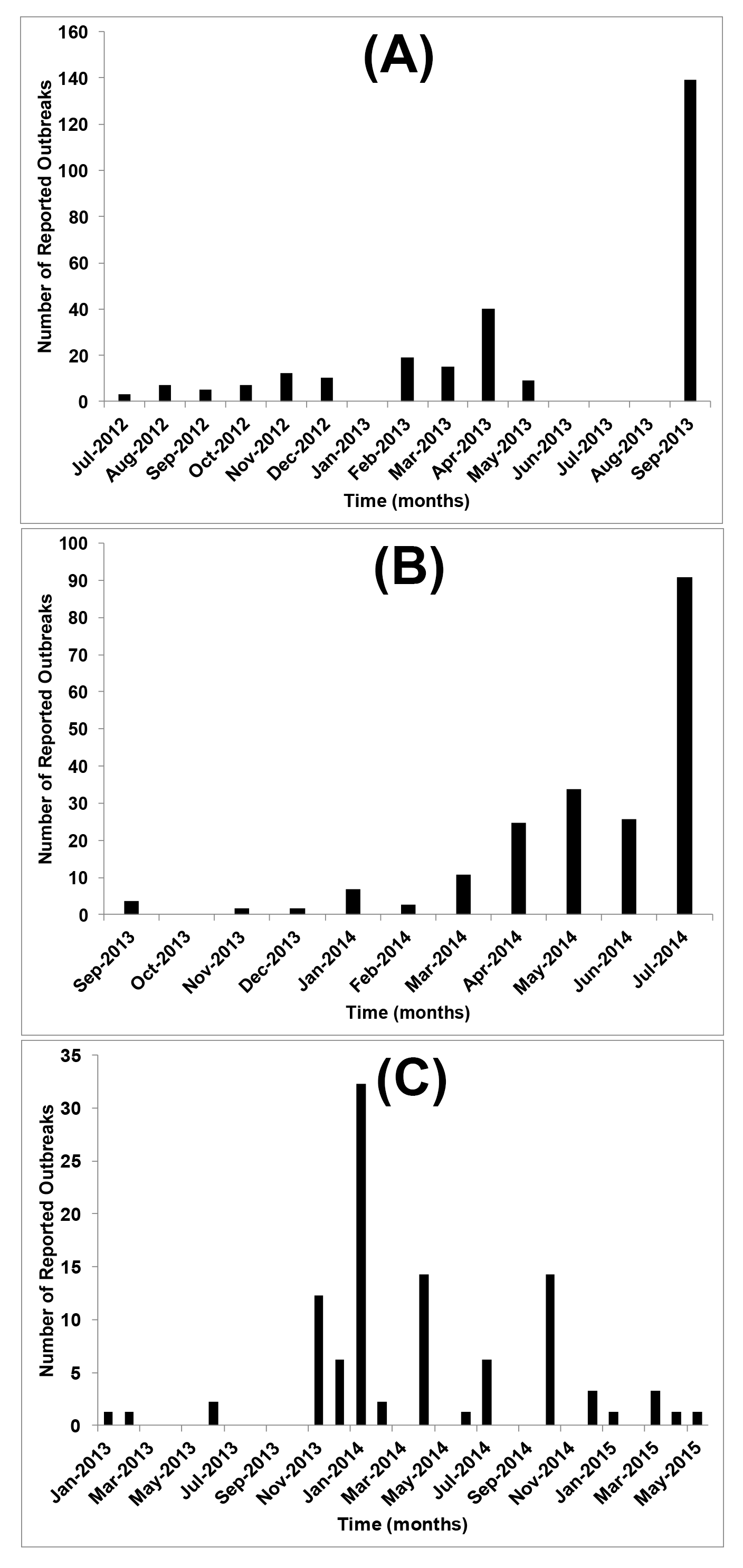
**

**Supplementary Figure 1.** Temporal distribution of lumpy skin disease outbreaks (per month per country) in cattle in the Middle East from July 2012 through May 2015. (A) represents outbreaks reported in Israel; (B) represents outbreaks reported in Turkey; (C) represents ME countries excluding Israel and Turkey
